# Identification of Key Pyroptosis-Related Genes and Distinct Pyroptosis-Related Clusters in Periodontitis

**DOI:** 10.3389/fimmu.2022.862049

**Published:** 2022-06-29

**Authors:** Wanchen Ning, Aneesha Acharya, Simin Li, Gerhard Schmalz, Shaohong Huang

**Affiliations:** ^1^ Stomatological Hospital, Southern Medical University, Guangzhou, China; ^2^ Dr. D. Y. Patil Dental College and Hospital, Dr. D. Y. Patil Vidyapeeth, Pune, India; ^3^ Department of Cariology, Endodontology and Periodontology, University Leipzig, Leipzig, Germany

**Keywords:** periodontitis, pyroptosis, inflammasome, immune microenvironment, prognostic

## Abstract

**Aim:**

This study aims to identify pyroptosis-related genes (PRGs), their functional immune characteristics, and distinct pyroptosis-related clusters in periodontitis.

**Methods:**

Differentially expressed (DE)-PRGs were determined by merging the expression profiles of GSE10334, GSE16134, and PRGs obtained from previous literatures and Molecular Signatures Database (MSigDB). Least absolute shrinkage and selection operator (LASSO) regression was applied to screen the prognostic PRGs and develop a prognostic model. Consensus clustering was applied to determine the pyroptosis-related clusters. Functional analysis and single-sample gene set enrichment analysis (ssGSEA) were performed to explore the biological characteristics and immune activities of the clusters. The hub pyroptosis-related modules were defined using weighted correlation network analysis (WGCNA).

**Results:**

Of the 26 periodontitis-related DE-PRGs, the highest positive relevance was for High-Mobility Group Box 1 (HMGB1) and SR-Related CTD Associated Factor 11 (SCAF11). A 14-PRG-based signature was developed through the LASSO model. In addition, three pyroptosis-related clusters were obtained based on the 14 prognostic PRGs. Caspase 3 (CASP3), Granzyme B (GZMB), Interleukin 1 Alpha (IL1A), IL1Beta (B), IL6, Phospholipase C Gamma 1 (PLCG1) and PYD And CARD Domain Containing (PYCARD) were dysregulated in the three clusters. Distinct biological functions and immune activities, including human leukocyte antigen (HLA) gene expression, immune cell infiltration, and immune pathway activities, were identified in the three pyroptosis-related clusters of periodontitis. Furthermore, the pink module associated with endoplasmic stress-related functions was found to be correlated with cluster 2 and was suggested as the hub pyroptosis-related module.

**Conclusion:**

The study identified 14 key pyroptosis-related genes, three distinct pyroptosis-related clusters, and one pyroptosis-related gene module describing several molecular aspects of pyroptosis in the pathogenesis and immune micro-environment regulation of periodontitis and also highlighted functional heterogeneity in pyroptosis-related mechanisms.

## Introduction

Periodontitis is a highly prevalent plaque biofilm-associated chronic inflammatory disease affecting the supporting structures of teeth that causes loss of attachment, including alveolar bone and connective tissues, as well as imposing significant systemic inflammatory burden (1, 2). At its core, the pathogenesis of periodontitis involves microbial dysbiosis in the subgingival dental plaque niche, which is followed by immune dysregulation, perturbed host response, and loss of homeostasis and ongoing inflammation, ultimately leading to tissue destruction (3). An increasing number of systemic conditions have been linked with periodontitis (4, 5), and multiple underlying mechanisms understood to mediate this association include circulating pro-inflammatory cytokines, bacterial cell components such as the keystone pathogen *Porphyromonas gingivalis* (*P. gingivalis*), among others (6). A large volume of research has focused on describing the molecular pathways mediating periodontal disease susceptibility, inflammation, and destruction (7), yet these molecular mechanisms are not completely delineated.

Programmed cell death (PCD), including apoptosis, necroptosis, NETosis, and pyroptosis, is recognized as a key feature of innate immune defense in infectious diseases (8, 9), whereby moderate degrees of pyroptosis may serve protective functions, but excessive degrees enable host-tissue destruction. The roles of different PCD pathways in periodontitis are not yet well established, and in particular, little is known about the role of pyroptosis (10, 11). Pyroptosis is a caspase (CASP)-activated form of PCD characterized by pore formation in plasma membranes, followed by swelling, cell lysis, and local release of pro-inflammatory mediators (12, 13). More recently, pyroptosis has been found to be driven by Gasdermin family proteins, chiefly including Gasdermin D (GSDMD), activated by pro-inflammatory CASPs located on inflammasomes (14). The Nod-Like Receptor (NLR) Family Pyrin Domain Containing 3 (NLRP3) inflammasome is a key canonical activator of CASP1 (15). Several studies have indicated that NLRP3 inflammasome production is a feature of periodontitis and reflected by increased levels of NLRP3-associated proteins in serum and saliva (16, 17). In the context of periodontitis, a number of recent *in vitro* and animal experimental studies have also documented the activation of GSDMD and NLRP3 inflammasome pathways, including that by *P. gingivali*s lipopolysaccharide (LPS) stimulation (18–23). *P. gingivali*s induced the activation of pyroptosis-related NLRP3 inflammasome, which is also implicated in atherosclerosis associated with periodontitis (24, 25). The inhibition of NLRP3 inflammasome in periodontitis is also documented as a mechanism of immune evasion (26, 27). Accruing evidence has shown that pyroptosis plays a role in periodontitis pathology, whereby several virulence factors associated with periodontal pathogens like *P. gingivalis* can trigger inflammasome activation *via* CASPs and downstream pyroptosis (28). CASP4/GSDMD initiated by bacterial LPS is shown to cause pyroptosis of periodontal ligament stem cells in periodontitis (29). *P. gingivalis* LPS is also found to mediate macrophage pyroptosis in periodontitis (30). However, very little is known about the detailed regulatory genetic and molecular machinery that underlies the involvement of pyroptosis in the pathogenesis and systemic sequelae of periodontitis.

Therefore, the current study was aimed to leverage publicly available transcriptomics datasets for integrative bioinformatics to identify a pyroptosis-related gene signature and its associated functional pathways in periodontitis, thereby deepening the understanding of putative pyroptosis-related mechanisms in periodontal diseases. The findings of this investigation could offer directions for future experimental research and uncover important pathogenic pathways and therapeutic targets for periodontitis.

## Materials and Methods

### Data Processing

Periodontitis-related datasets from affected gingival tissues and healthy controls were obtained from the Gene Expression Omnibus (GEO) database of NCBI and included GSE10334 (28) and GSE16134 (31) (**Supplementary Table S1**). A total of 37 pyroptosis-related genes (PRGs) were obtained, including 16 PRGs from previous literature (32) and 24 PRGs from the Molecular Signatures Database (MSigDB) (https://www.gsea-msigdb.org/gsea/msigdb/), excluding three overlapped PRGs. Next, to obtain the periodontitis-related PRGs, the above 37 PRGs were merged with the expression profiles of GSE10334 and GSE16134.

### Identification of the Differentially Expressed Periodontitis-Related PRGs

Differential expression analysis was performed to screen periodontitis-related PRGs with GSE10334 and GSE16134 each, using the “Linear Models for Microarray data” (“limma”) package in R (33) with Benjamini–Hochberg false discovery rate (34) adjustment and with a *P*-value <0.05 and |log FC| > 0 as the threshold to screen differentially expressed (DE)-PRGs. These were then visualized in a heat map. The expression pattern of the PRGs in disease and healthy controls was identified in the GSE10334 dataset, further validated in the GSE16134 dataset, and displayed in box plots using the “limma” package in R (33). A protein–protein interaction (PPI) network was constructed to assess the gene interactions among the 26 DE-PRGs using the Search Tool for the Retrieval of Interacting Genes database (https://string-db.org/), visualized with "igraph" package in R (http://igraph.org). To evaluate the correlation between PRG pairs, Pearson’s correlation coefficient (35) was computed for the DE-PRGs in periodontitis samples from GSE10334 and visualized using “corrplot” in R (36).

### Construction and Validation of a Prognostic Model Based on Periodontitis-Related PRGs

The significant DE-PRGs in GSE10334 were selected by univariate logistic regression analysis (*P*-value <0.05) and visualized in forest plots. A prognostic model was constructed to select the most relevant DE-PRGs using the least absolute shrinkage and selection operator (LASSO) regression, selecting the penalty parameter (*λ*) with tenfold cross-validation. Subsequently, multivariate logistic regression analysis was applied to develop a diagnostic model based on the selected prognostic signatures. The risk scores of each sample in the training set (GSE10334) and the test set (GSE16134) were calculated based on the following risk score formula: $\text{risk score}=\sum_{i}^{7}\left(X_{i}\times Y_{i}\right)$ (X: coefficients, Y: gene expression level) (37). The risk scores were visualized using box plots, and analysis of variance (ANOVA) was applied. The predictive accuracy of the training set and the test set was evaluated by plotting a receiver operating characteristic (ROC) curve (38) with the “pRPC” package (39) in R.

### Consensus Clustering Based on the Prognostic DE-PRGs

The pyroptosis-related clusters in 183 periodontitis samples (GSE10334) were determined using “ConsensusClusterPlus” package in R (40) based on the prognostic DE-PRGs. The cluster number was determined according to the consensus matrix, the consensus index-cumulative distribution function (CDF) curve, and the delta area score of CDF. The gene distribution of different clusters was evaluated by principal component analysis (PCA). In addition, the gene expression profiles of the prognostic DE-PRGs within different clusters were compared using Student’s *T*-test, where DE-PRGs with a *P*-value <0.001 were considered as the specific prognostic PRGs in periodontitis.

### Functional Analysis of Different Clusters

The DEGs in different clusters were filtered with the criteria of adjusted *P*-value <0.05 and |log FC| ≥0.2. The enriched functions for these DEGs were explored and compared by investigating their enriched Gene Ontology (GO) terms, including biological processes (BP), cellular components (CC), and molecular functions (MF), as well as Kyoto Encyclopedia of Genes and Genomes (KEGG) pathway analysis, using “clusterProfiler” package in R and visualized using bubble plots.

### Single-Sample Gene Set Enrichment Analysis for Different Clusters

The immune scores of human leukocyte antigen (HLA)-related gene expression, infiltrating immune cells, and immune pathways in periodontitis as well as in different clusters were calculated and compared by the single-sample Gene Set Enrichment Analysis (ssGSEA) (41) and depicted using box plots.

### Weighted Correlation Network Analysis to Identify the Hub Pyroptosis-Related Modules

The weighted correlation network analysis (WGCNA) package (42) was applied to the DEGs in the clusters to construct a co-expression network and identify pyroptosis-related gene modules. Briefly, Pearson’s correlation coefficients were firstly calculated, an adjacency matrix was built, and the optimum power (*β*) was selected to construct a scale-free topology. Then, average linkage hierarchical clustering was conducted to identify modules through topological overlap matrix approach (cutHeight = 20, 000, minModuleSize = 60). Next, module eigengenes, representing the correlation between different clusters and modules, were applied to select significant modules. A high correlation between gene significance (GS) and module membership (MM) implied an intra-modular hub gene module. Subsequently, functional enrichment analysis was performed to identify the significant GOs, BPs, MFs, and KEGG pathways enriched by the hub pyroptosis-related gene module using the “clusterProfiler” package in R (43).

## Results

### Identification of DE-PRGs in Periodontitis

**Figure 1:**
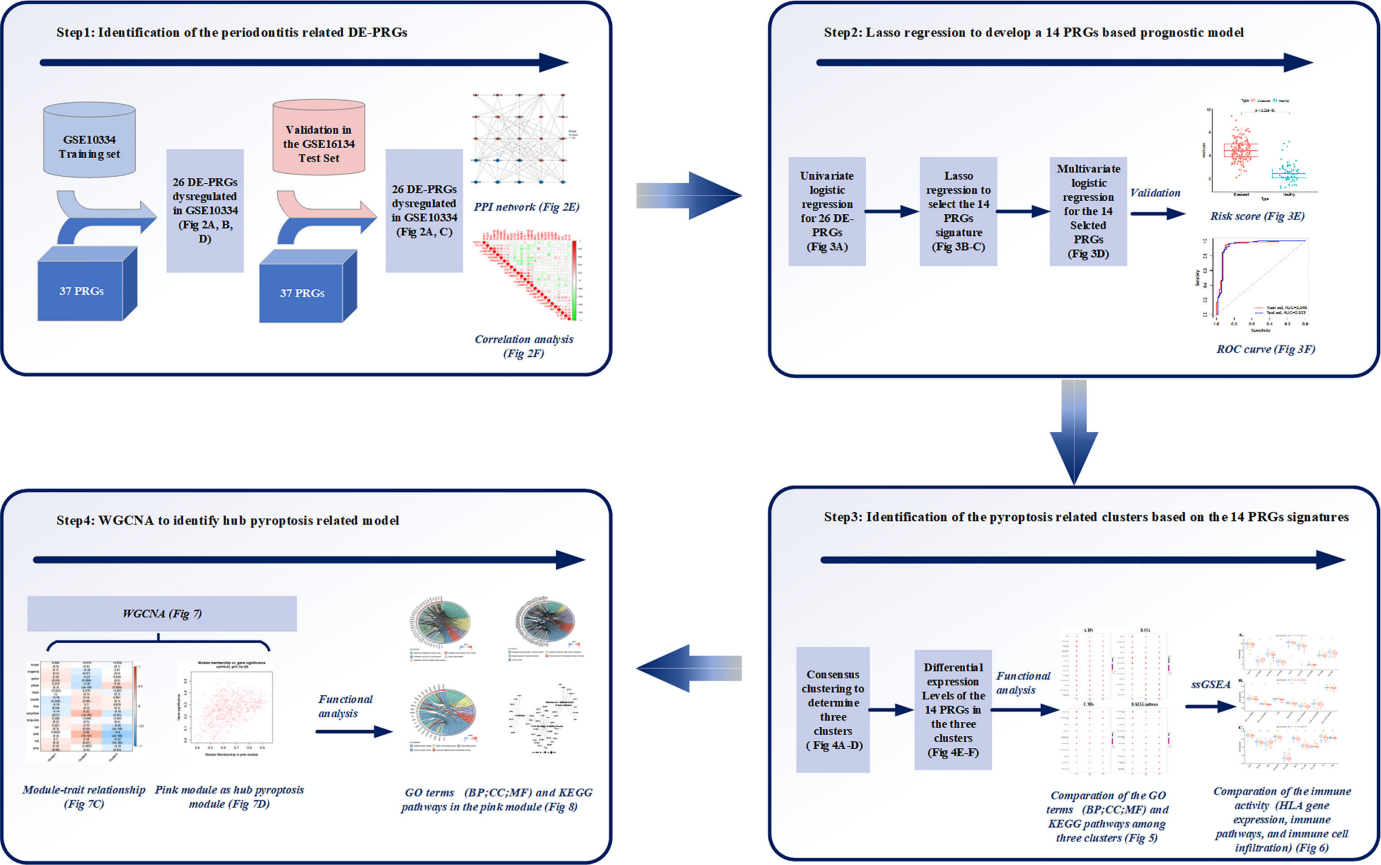
Study workflow. Step1: 26 differentially expressed (DE)-pyroptosis-related genes (PRGs) were determined by merging the expression profiles of GSE10334 and 37 obtained PRGs. The 26 DE-PRGs were further validated in another periodontitis dataset (GSE16134). Protein-protein interaction (PPI) network and correlation analysis were conducted to investigate the interaction and correlation between the 26 DE-PRGs; Step2: The prognostic value of the 26 DE-PRGs in periodontitis (Training set) was assessed by univariate logistic regression analysis, and least absolute shrinkage and selection operator (LASSO) regression was applied to screen 14 prognostic PRGs from the 26 DE-PRGs. Then, a 14-PRGs signature model was developed based on multivariate logistic regression analysis. The risk scores and receiver operating characteristic (ROC) curves were applied for the model validation; Step3: Consensus clustering was applied based on the 14 signatures, and three pyroptosis-related clusters were determined. Differential expression levels of the 14 PRGs were compared among the three clusters. Functional analysis was applied to compare the Gene Ontology (GO) terms, including Biological Processes (BP), Cellular Components (CC), and Molecular Functions (MF), as well as Kyoto Encyclopedia of Genes and Genomes (KEGG) pathways among the three clusters. Single-sample gene set enrichment analysis (ssGSEA) were applied to compare the immune activities among the three clusters. Step4: Weighted correlation network analysis (WGCNA) identified the pink module as the hub pyroptosis-related module based on the module-trait relationship. Functional analysis was conducted for the pink module, in order to explore the role of pyroptosis in periodontitis

**Figure 2:**
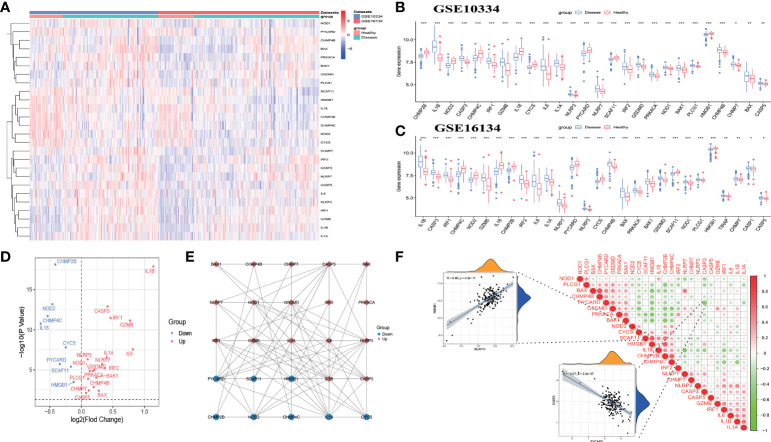
Identification of differentially expressed pyroptosis-related genes (DE-PRGs) in periodontitis. **(A)** Heat map of 26 DE-PRGs in the periodontitis dataset (GSE10334 and GSE16134). **(B, C)** The box plots show the different expression levels of the 26 PRGs between disease (periodontitis) and healthy samples in the GSE10334 dataset **(B)** and GSE16134 dataset **(C)**. *P < 0.05, **P < 0.01, ***P < 0.001. **(D)** Volcano plot of the 26 DE-PRGs in the GSE10334 dataset. **(E)** Protein–protein interaction (PPI) network of the 26 DE-PRGs. **(F)** Correlations of the 26 DE-PRGs in the periodontitis samples Red, positive correlation; Green, negative correlation. The color depth and the size reflect the strength of the relevance. The strongest positive and the strongest negative correlation were displayed in scatter plots.

The workflow of the study is displayed in **Figure 1**. Of the 37 PRGs, a total of 26 DE-PRGs were identified from the GSE10334 dataset (train set), and the same upregulated and downregulated DE-PRGs were determined in the GSE16134 dataset (test set) (**Figures 2A–D**). Of these, 18 PRGs were upregulated in periodontitis, including interleukin (IL) 1 beta (B), IL6, NLRP3, NLRP7, nucleotide binding oligomerization domain containing 1 (NOD1), phospholipase C gamma 1 (PLCG1), protein kinase CAMP-activated catalytic subunit alpha (PRKACA), BCL2 antagonist/killer 1 (BAK1), BCL2-associated X (BAX), CASP3, CASP5, charged multivesicular body protein (CHMP) 4B, CHMP7, GSDMD, granzyme B (GZMB), IL1A, interferon regulatory factor 1 (IRF1), and IRF2, while eight PRGs were downregulated in periodontitis, including IL18, CHMP2B, NOD2, CHMP4C, cytochrome C, somatic (CYCS), PYD and CARD domain containing (PYCARD), SR-related CTD associated factor 11 (SCAF11), and high mobility group box 1 (HMGB1) (**Figures 2A–D**). The PPI network shows the interactions between the 26 periodontitis-related PRGs (**Figure 2E**). The correlation analysis presented the relevance of PRG pairs in periodontitis (**Figure 2F**). The highest positive correlation was noted between HMGB1 and SCAF11 (*R* = 0.46, *p* = 4.3e-11), while the strongest negatively correlated pair were CASP3 and PYCARD (*R* = -0.37, *p* = 2.2e-07) (**Figure 2F**).

### Development and Validation of the LASSO Model

**Figure 3:**
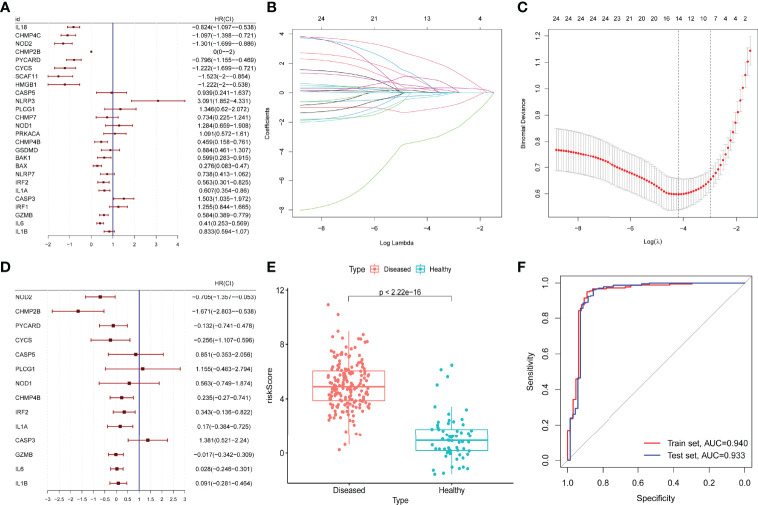
Construction and validation of a prognostic model. **(A)** Univariate logistic regression analysis for the 26 differentially expressed pyroptosis-related genes (PRGs) in periodontitis samples (GSE10334) with *P* < 0.05. **(B, C)** A total of 14 prognostic genes were selected among 26 periodontitis-related PRGs with least absolute shrinkage and selection operator (LASSO) model, selecting the optimum parameter (*λ*) as 14 with 10-fold cross-validation. **(D)** Multivariate logistic regression for the 14 prognostic PRGs. **(E)** Risk scores of the disease (periodontitis) and healthy samples in the GSE10334 dataset (*P* < 2.2E-16). **(F)** Receiver operating characteristic (ROC) curve of the GSE10334 dataset (training set) and the GSE16134 dataset (test set). The area under the curve (AUC) value of the train set and the test set is 0.940 and 0.933.

The prognostic value of the 26 DE-PRGs in periodontitis (train set) was assessed by univariate logistic regression analysis (**Figure 3A**). All the 26 DE-PRGs were included in the LASSO model, with a *P*-value <0.001 (**Figure 3A**). Then, the optimum parameter (*λ*) was selected as 14 (**Figures 3B, C**), and a 14-PRG signature model was developed based on a multivariate logistic regression analysis (**Figure 3D**). The risk scores of the disease samples were significantly higher than those of the healthy samples in both the training set (**Figure 3E**) and the test set (*P* < 2.2E-16). The accuracy and area under the curve of the training set and the test set was 0.940 and 0.933, respectively (**Figure 3F**), suggesting that the model has “outstanding” robustness (38).

### Three Clusters Were Determined Based on the 14 Prognostic DE-PRGs

**Figure 4:**
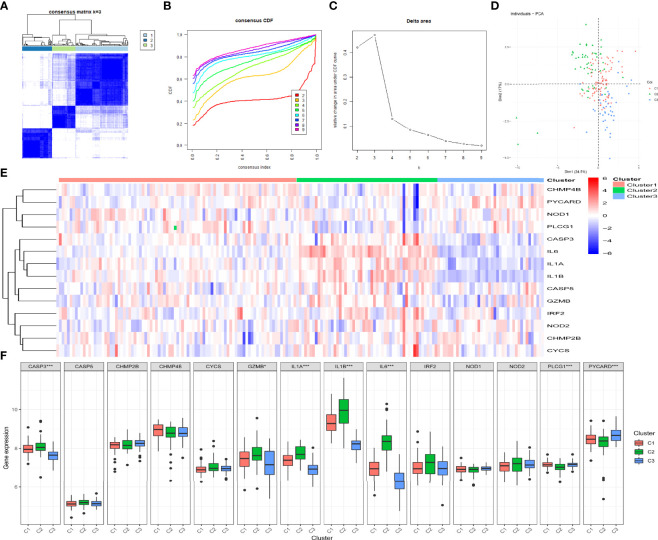
Identification of the pyroptosis-related clusters in periodontitis. **(A–C)** Consensus clustering for the 183 periodontitis samples in GSE10334 based on the prognostic pyroptosis-related genes (PRGs). Three clusters were classified according to the consensus matrix **(A)**, consensus index of cumulative distribution function (CDF), and CDF delta area curve **(C)** for *k* = 3 by increasing the index from 2 to 9. **(D)** The principal component analysis (PCA) shows a different distribution of the three clusters. **(E, F)** The expressions of the 14 prognostic PRGs in the three clusters are shown in the heat map **(E)** and box plots **(F)**. *P < 0.05, **P < 0.01, ***P < 0.001.

By determining the clustering variable (*k*) as 3 (**Figures 4A–C**), the 183 periodontitis samples in the GSE10334 dataset were classified into three pyroptosis-related clusters based on the 14 prognostic PRGs, including 91 cases in cluster 1 (C1), 52 cases in cluster 2 (C2), and 40 cases in cluster 3 (C3). The PCA indicated a difference among the three clusters (**Figure 4D**). The heat map displayed the gene expression profiles of the 14 prognostic PRGs in the three clusters (**Figure 4E**). Of these, the expression levels of CASP3, GZMB, IL1A, IL1B, and IL6 were highest in C2, whereas these genes presented lowest in C3 (*P*-value <0.001, excluding GZMB with P-value <0.05) (**Figure 4F**). On the contrary, PLCG1 and PYCARD presented the lowest level in C2 but the highest level in C3 (*P*-value < 0.001) (**Figure 4F**).

### Distinct Biological Functions Enriched in the Three Clusters

**Figure 5:**
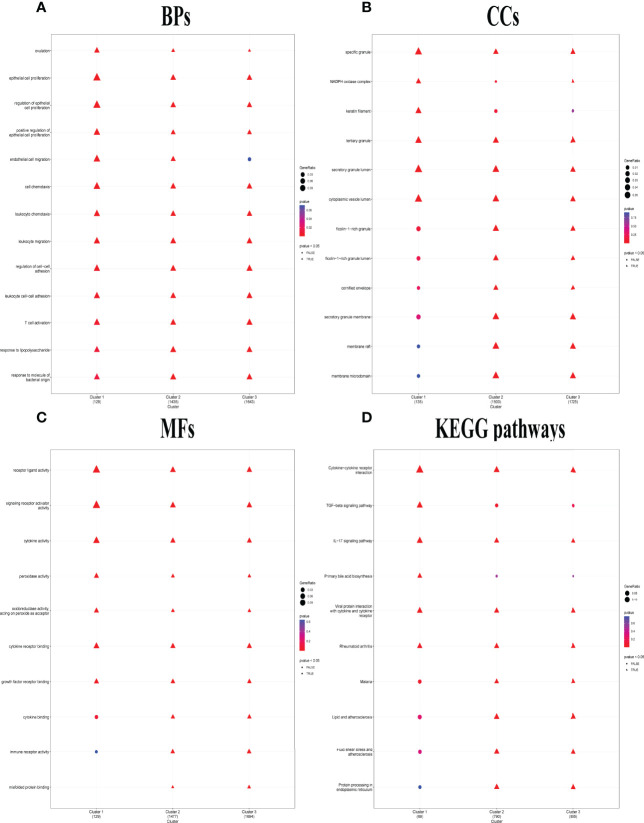
Distinct biological functions identified among the three pyroptosis-related clusters. **(A–C)** Comparison of the GO-BPs **(A)**, GO-CCs **(B)**, and GO-MFs **(C)** enriched in the three clusters. GO, Gene Ontology; BP, biological processes; CC, cellular components; MF, molecular functions. **(D)** Kyoto Encyclopedia of Genes and Genomes (KEGG) pathways enriched in the three clusters. The terms with *p*-value <0.05 were shown as triangle points (TRUE), and others were displayed as round points (FALSE).

Distinct enriched functional GO terms, including BP, CC, MF, and KEGG pathways, were identified in the three clusters, particularly in C1 (**Figure 5**). Among the GO-BP terms, C1 and C2 were distinguished from C3 by significant enrichment in endothelial cell migration (**Figure 5A**). Among GO-CC terms, C1 and C3 were significantly enriched in NADPH oxidase complex, while C1 alone was enriched in keratin filament but not enriched in ficolin-1-rich granule, ficolin-1-rich granule lumen, cornified envelope, secretory granule membrane, membrane raft, and membrane microdomain (**Figure 5B**). Considering GO-MF, C1 distinctly lacked enrichment in cytokine binding, immune receptor activity, and misfolded protein binding (**Figure 5C**), whereas transforming growth factor (TGF) beta signaling pathway and primary bile acid biosynthesis were only enriched in C1. KEGG pathways including malaria, lipid and atherosclerosis, fluid shear stress and atherosclerosis, and protein processing in endoplasmic reticulum lacked enrichment only in C1 (**Figure 5D**).

### Distinct Immune Characteristics Characterized Both Periodontitis and the Three Pyroptosis-Related Clusters

Significant differences were noted in the relative enrichment scores of HLA gene expression, immune cell infiltration, and immune pathway activities between periodontitis (diseased) and healthy tissues, indicating that the immune micro-environment acts as a vital regulator in periodontitis pathology (**Supplementary Figure S1**). Higher levels of HLA-related genes, such as HLA-DP alpha 1 (DPA1), HLA-B, and HLA-C (**Supplementary Figure S1A**), more infiltrating immune cells, such as B cells, CD8+ T cells, and neutrophils (**Supplementary Figure S1B**), and higher activation of immune pathways, such as type II interferon (IFN) response, inflammation promoting, and chemokine receptors (CCR) (**Supplementary Figure S1C**), were detected in periodontitis compared to healthy controls. No significant difference in the levels of HLA-DQ alpha 1 (DQA1), HLA-DP beta 2 (DPB2), T helper 2 (Th2) cells, major histocompatibility complex class I, and type I IFN response were detected between periodontitis and controls (**Supplementary Figure S1**).

**Figure 6:**
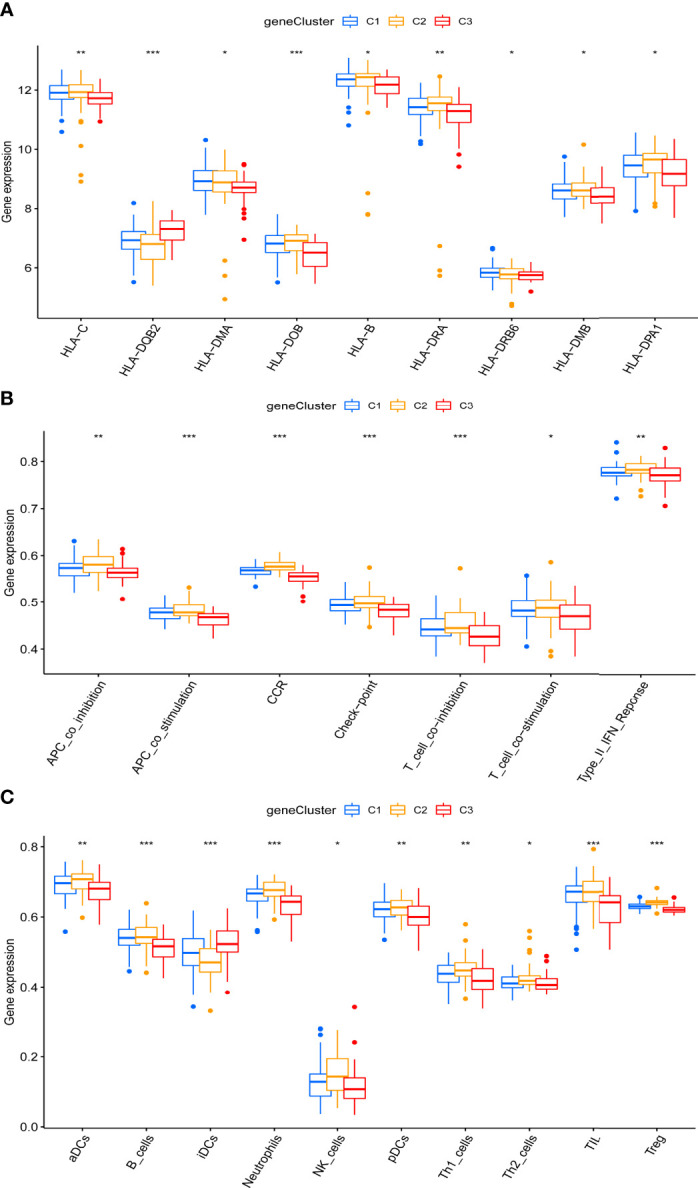
Distinct immune characteristics underlined in the three pyroptosis-related clusters. **(A–C)** Human leukocyte antigen (HLA) gene expression **(A)**, immune pathway activities **(B)**, and immune cell infiltration **(C)** of the three pyroptosis-related clusters. *P < 0.05, **P < 0.01, ***P < 0.001.

Importantly, marked differences were observed in the immune microenvironment among the three pyroptosis-related clusters, indicating a close relationship between pyroptosis and immune regulation. Compared to C1 and C2, C3 presented higher levels of HLA-DQ beta 2 (DQB2) but lower levels of other HLA-related genes, such as HLA-C, HLA-DM alpha (DMA), HLA-DO beta (DOB), HLA-B, HLA-DR alpha (DRA), HLA-DR beta 6 (DRB6), HLA-M beta (DMB), and HLA-DPA1 (**Figure 6A**). Unlike C1 and C2, more infiltrating iDCs—whereas fewer infiltrating aDCs, B cells, neutrophils, natural killer cells, pDCs, Th1/TH2 cells, tumor-infiltrating lymphocytes cells, and Treg cells were likewise observed in C3 (**Figure 6C**). Specifically, compared with C1 and C2, C3 showed a lower activation of immune pathways, including antigen-presenting cell (APC) co-inhibition, APC co-stimulation, CCR, check-point, T cell co-inhibition, T cell co-stimulation, and type II IFN response (**Figure 6B**).

### Identification of the Hub Pyroptosis-Related Gene Module

**Figure 7:**
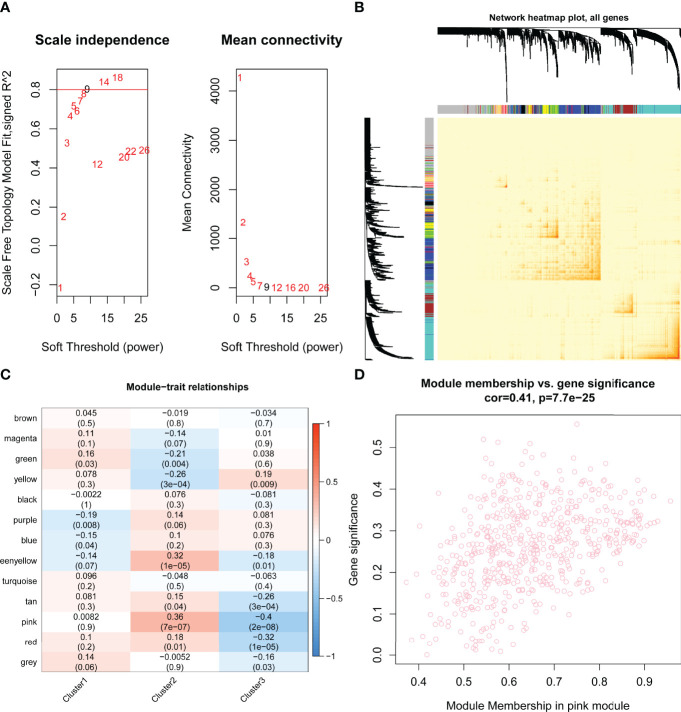
Identification of hub pyroptosis-related gene modules by performing weighted gene correlation network analysis (WGCNA). **(A)** Scale independence and mean connectivity analysis for various soft threshold powers, where the optimum power = 9. **(B)** Network heat map plot of topological overlap for all genes. Each row and column correspond to a gene. The color row underneath the dendrogram indicated the module assignment, of which 13 modules were identified according to the dynamic tree cut. **(C)** The module–trait relationships between the 13 modules and three clusters are shown in the heat map. The most significant relationship was between the pink module eigengene and cluster 2 (Cor = 0.36, *P* = 7e-07). **(D)** The scatter plots of module membership in pink module vs. gene significance in cluster 2 (Cor=0.41, P=7.7e-25). Cor, correlation..

By WGCNA, 13 gene modules were determined based on a dynamic tree (**Figures 7A, B** **)**. Based on the module–trait relationships between 13 modules and three clusters, the most significant correlation was seen between the pink module and cluster C2 (Cor = 0.36, *P* = 7e-07) (**Figure 7C**). The association of the module membership in the pink module with gene significance in C2 was visualized in the scatter plots (Cor=0.41, P=7.7e-25) (**Figure 7D**).

### Biological Functions Enriched in the Pink Module

**Figure 8:**
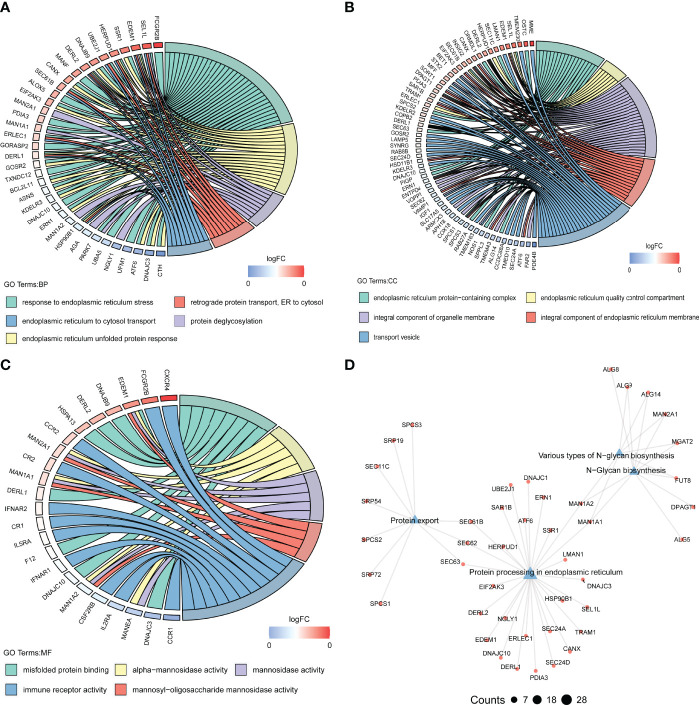
Biological functions behind the pink module. **(A–C)** GO-BPs **(A)**, GO-CCs **(B)**, and GO-MFs **(C)** enriched in the pink module. GO, Gene Ontology; BP, biological processes; CC, cellular components; MF, molecular functions. **(D)** Kyoto Encyclopedia of Genes and Genomes (KEGG) pathways enriched in the pink module. The terms with *p*-value <0.05 are visualized.

To explore the functional mechanisms indicated by the pyroptosis-mediated pink gene network module, the GO characters (BP, CC, and MF) as well as KEGG pathways were investigated. Most genes in the pink module were found to be enriched in BPs such as response to endoplasmic reticulum stress and endoplasmic reticulum unfolded protein response (**Figure 8A**), in CCs such as integral component of organelle membrane and transport vesicle (**Figure 8B**), and in MFs including immune receptor activity, misfold protein binding, and mannosidase activity (**Figure 8C**). The KEGG pathway analysis showed that the pink module genes were mainly enriched in pathways including protein export pathway, protein process in endoplasmic reticulum pathway, various types of N-glycan biosynthesis pathway, and N-glycan biosynthesis pathway (**Figure 8D**).

## Discussion

The present study used a suite of comprehensive bioinformatics analyses to describe multiple molecular aspects of pyroptosis in the pathogenesis of periodontitis by leveraging publicly available transcriptome data. By applying clustering approaches, hub pyroptosis-related genes and their key functional roles in periodontitis were identified. At the first step, PRGs significantly dysregulated in periodontitis were determined. Among the upregulated PRGs, high log fold changes were evident for the pro-inflammatory mediators IL1B, IL6, and GZMB. The inflammasome-mediated activation of CASPs, such as CASP1, CASP11, or CASP8, is known to cleave GSDMD to induce the maturation of the pro-inflammatory cytokine IL1B (44–47). GZMB, a serine protease, is shown to cleave gasdermin E to induce pyroptosis (48). GZMB is associated with chronic inflammation in multiple conditions, in particular, destructive skin and connective tissue conditions, and induces extracellular matrix destruction and pro-inflammatory cytokine activation (49), but experimental evidence concerning its role in periodontitis-associated pyroptosis is scarce. Among the DE-PRGs, CASP3 was notable in terms of high log fold change. Increased CASP3 levels have been observed in gingival crevicular fluid from periodontitis (50) and also seen to decline in the short term following therapy (51). Mechanistically, the short-chain fatty acid butyrate has been found to induce CASP3-mediated pyroptosis in gingival epithelial cells (52). Downregulated DE-PRGs with high fold change included NOD2, CHMP2B, and CHMP4C. NOD2 is crucial for bacterial pathogen recognition and its subsequent macrophage response in periodontal tissue, and in agreement with our findings, its knockout was shown to reduce bone resorption in a mouse model of *Aggregatibacter actinomycetemcomitans (Aa)*-induced periodontitis (53). The CHMP proteins are part of the endosomal sorting complex required for transport (ESCRT) III machinery involved in regulating endosomal transport and cargo recognition (54, 55) and are crucial to the repair of plasma membrane damage and resistance to pyroptosis (56), but experimental evidence specifically investigating the role of CHMP family of proteins in periodontitis-related pyroptosis is lacking. Of note is that the expression pattern of NLRP3-associated genes, such as ASC (PYCARD), and inflammasome-associated cytokines has shown different expression patterns in literature regarding periodontitis pathology (16, 57). Aral et al. (57) showed that *P. gingivalis* exposure of human gingival fibroblasts along with ATP led to the downregulation of ASC and NLRP3 while upregulating IL-1, whereas, in contrast, *Fusobacterium nucleatum (F.nucleatum)* infection was marked by increased levels of NLRP3, ASC, and IL-1B. Similar findings of *P. gingivalis*-containing biofilm infection leading to the downregulation of NLRP3 inflammasome were earlier reported by Belibasakis et al. (58), indicating that the pathogen-induced dampening of innate immune response to favor pathogen persistence might underlie this finding. Furthermore, the downregulation of inflammasome regulators in periodontal disease has also been noted by Aral et al. (59) who found that ASC/PYCARD was negatively correlated to probing depth, although no differences were noted between ASC/PYCARD between disease groups. In the present study, lower levels of IL-18 were noted in the disease group as compared to the healthy controls, which is suggestive of the dichotomous roles of IL-18. Lower levels of IL-18 have been noted in early periodontal disease as compared to healthy controls (60). Clinically, periodontal therapy has been found to reduce NLRP3 but not IL-18 (61). Distinct and independent processes in NLRP3 inflammasomes *in vitro* are shown to elicit IL-1B and IL-18 production in response to non-canonical stimulation, which can fine-tune the pyroptosis response (62) and is likely to account for the opposing patterns observed in periodontitis samples. The role of non-canonical NLRP3 inflammasome activation in periodontitis needs further study.

Among the PRG pairs, HMGB1 and SCAF11 showed the strongest positive correlation in periodontitis. HMBG1 is stimulated by inflammatory stimuli, can act on toll-like receptors, and can also enhance chemotaxis, depending on the redox state (63). In periodontitis, HMBG1 secretion due to bacterial stimulation has been documented and shown to prolong the inflammatory responses (64). SCAF11 is involved in mRNA splicing and recently recognized as implicated in several types of cancer, but its involvement in periodontitis remains to be clarified. The strongest negative correlation was noted between CASP3 and PYCARD, while CASP3 and NLRP3 were both also negatively correlated with IL-18. CASP3 is common to both apoptosis and pyroptosis pathways, whereas PYCARD is a key inflammasome regulator (65). These findings could suggest that pyroptosis *via* the non-canonical NLRP3 pathway activation could occur in periodontitis, plausibly occurring in a distinct temporal stage or in differing disease characteristics. In support, ATP is shown to induce pyroptosis in macrophages through the alternative CASP3 pathway when the canonical NLRP3 pathway is blocked by pathogens as a mechanism to counter pathogen evasion (66). The present findings are closely aligned with an earlier study which showed that PYCARD/ASC knockout led to the attenuation of the canonical pyroptosis-associated CASP1 but increased the number of non-canonical pyroptosis-associated CASP3- and CASP8-expressing gastric epithelial cells (67), which occurred independent of mucosal inflammation.

The 14-PRG signature for periodontitis achieved excellent prediction accuracy in disease classification and provides a basis for translational research. To further explore the mechanistic aspects, consensus clustering was applied and demonstrated three distinct clusters, whereby C2 was characterized by CASP3, GZMB, IL1A, IL1B, and IL6, representing CASP3-stimulated pyroptosis pathway dominance. On the other hand, C3 was marked by PLCG1 and PYCARD, where lipid peroxidation-mediated PLCG1 activation can drive GSDMD and induce pyroptosis (68), and PYCARD is a NLR component implicated in reactive oxygen species-mediated pyroptosis involving NLRP3 activation (69, 70). The potential relevance of these differences in pyroptosis mechanisms between clusters may represent the molecular subtypes of the disease in the context of pyroptosis and warrants further investigation in experimental research. The functional analysis indicated that C1 was enriched in endothelial cell migration, NADPH oxidase, keratin filament, and TGF beta signaling pathway, suggesting the dominance of canonical pathway-mediated pyroptosis. Pyroptosis can be mediated by the canonical inflammasome pathway *via* CASP1 that stimulates the IL18/INFγ/NADPH oxidase axis and can be triggered by gram-negative bacteria (71–73). Cluster C3 was marked by higher levels of HLA-DQB2, an increase in infiltrating iDCs, and lower activation of APC and T cell-related immune pathways, along with a lower type II IFN response. Dendritic cells display multiple modes of inflammasome activation upon encountering a microbial product or endogenous triggers, resulting in either pyroptosis or a hyperactive state that stimulates adaptive immunity and T cell activation (74). Bacterial LPS is shown to induce CASP11-mediated non-canonical inflammasome activity, which leads to pyroptosis (75). Furthermore, while PMN infiltration was higher in periodontitis as compared to healthy tissues, C3 manifested a relatively lower PMN signature as compared to C1 and C2. Considering together with the findings that C3 was marked by higher PYCARD and iDCs but lower CASP3, GZMB, and pro-inflammatory cytokines, this raises further questions regarding the clinical and microbiological correlates of this cluster. It is also plausible that C3 may represent an earlier temporal stage of periodontitis or periodontal pathogen-associated immune evasion. Incidentally, the periodontal pathogen *P. gingivalis* has been associated with impairment of PMN recruitment (76). Increased HLA-DQB2 expression has been associated with susceptibility to the autoimmune disease rheumatoid arthritis (77) and increased renal transplant rejection (78). Therefore, the cluster C3 could also represent distinct host susceptibility associated with deregulation of immune tolerance, including pyroptosis. Of note is that non-canonical pyroptosis pathway activation has been associated with autoimmune disease (79), and our earlier research has shown distinct immune subtypes to exist in periodontitis (80). The role of pyroptosis should be investigated in these contexts. Together with the data from earlier investigation showing PYCARD suppression that led to increased CASP3 and CASP8 in gastric epithelial cells (67), these findings suggest the possibility that the cluster C3 represented a greater non-canonical inflammasome pathway molecular activity as compared to C1 and C2.

Future research should also address variations in periodontitis-associated biofilm composition and functions linked to specific pyroptosis pathways. Importantly, in the current study, metadata regarding clinical disease characteristics, such as disease severity or associated risk factors, were unavailable for correlation with pyroptosis-based clusters and should be addressed in future studies. The nature of host inflammatory responses and associated cell death varies with disease. In case of apical periodontitis, it has been shown that pyroptosis increases with the progression of the disease (81). In periodontitis, recent work has highlighted that genes associated with apoptosis and hypoxia show the largest changes during the disease initiation stage of 2 weeks; autophagy-related genes show maximum changes during disease progression stages and are associated with distinct oral microbiome features (82). Similar research into the temporal progression of disease or its severity and its association with pyroptosis is essential. CASP8, which can cleave GSDMD, resulting in pyroptosis activation (83), has been shown to be expressed at lower levels in aggressive periodontitis as compared to chronic periodontitis (84). An increase in CASP3 expression with disease has also been documented (85); however, whether the C3 samples manifested a lower severity in conjunction with a low CASP3 expression or whether disease progression patterns were distinct could not be ascertained in the present analysis. Additionally, several PRGs are implicated in other forms of cell death, and the balance of pyroptosis with other mechanisms of PCD, which occurs synergistically, also merits deeper investigation.

WGCNA showed a high correlation of the pink module with C2, which was found to be enriched in endoplasmic reticulum (ER) stress, vesicle transport, protein export, unfolded protein response, N-glycan biosynthesis pathway, and related functions. Increased ER stress in periodontitis has been noted (86). LPS has been shown to induce the ER stress response, an adaptive mechanism which, when excessive, leads to NLRP3 inflammasome activation and pyroptosis *via* the CASP1 pathway (87). *P. gingivalis* LPS has been shown to stimulate ER stress and, consequently, apoptosis in human umbilical vein endothelial cells (88), but the effects on pyroptosis are not established. In periodontitis, long-standing inflammation has been shown to induce ER stress *via* lysine acetyltransferase 6B (89). Others have noted that the ER stress-induced alveolar bone resorption in periodontitis was independent of inflammatory cytokine release (90). Taken together, these data suggest that multiple pathways of pyroptosis may be implicated in periodontitis with heterogeneous occurrence; furthermore, distinct molecular subtypes may exist in terms of their relative dominance.

These data highlight the necessity of further studies to unravel the mechanisms of pyroptosis involvement in periodontitis. Such research assumes significance considering the possibility of therapeutic interventions directed at pyroptosis blockade (91, 92) that can offer a novel management modality for periodontitis and syndemic oral-systemic conditions. The significance of pyroptosis in the linkage of periodontitis-associated systemic disease has been recently demonstrated in the context of rheumatoid arthritis using bioinformatics analysis (93), and additional studies are warranted. The findings of this study should be addressed in the context of its limitations. The 14-PRG signature was not verified with clinical or experimental data. In addition, the GEO datasets analyzed in the present study were of a modest sample size, which further necessitates verification experiments. As such, the presented findings must be considered as preliminary and the basis for directing future research.

## Conclusion

The present study identified a highly robust, 14-PRG signature of periodontitis. Furthermore, three distinct pyroptosis-related clusters were identified, with differences in enriched functional biological functions and immune microenvironments indicated by HLA gene expression, immune cell infiltration, and immune pathway patterns. A hub pyroptosis-related module associated with ER stress and related functions was closely representative of cluster 2. These findings presented several functional aspects of pyroptosis involvement in periodontitis and also suggested heterogeneity in the relatively dominant pyroptosis-related pathways, which necessitates future investigation in the context of disease features, susceptibility, and longitudinal progression patterns.

## Data Availability Statement

The datasets presented in this study can be found in online repositories. The names of the repository/repositories and accession number(s) can be found in the article/**Supplementary Material**.

## Author Contributions

WN designed the overall study workflow, analyzed the data, prepared the figures and tables, authored and reviewed drafts of the manuscript, and approved the final draft. AA, and SL aided in data analyses, prepared the figures and tables, were involved in proofreading and deep editing, and approved the final draft. GS devised the main conceptual idea, supervised the whole work, and approved the final draft. SH devised the main conceptual idea, supervised the whole work, and approved the final draft. All authors contributed to the article and approved the submitted version.

## Funding

We appreciate the research funding provided by the Science Research Cultivation Program of Stomatological Hospital, Southern Medical University (grant no. PY2021002 for WN and grant no. PY2020004 for SL). We appreciate the research funding provided by International Postdoctoral Exchange Fellowship Program (Talent-Introduction Program) (grant no. YJ20210260 for WN).

## Conflict of Interest

The authors declare that the research was conducted in the absence of any commercial or financial relationships that could be construed as a potential conflict of interest.

## Publisher’s Note

All claims expressed in this article are solely those of the authors and do not necessarily represent those of their affiliated organizations, or those of the publisher, the editors and the reviewers. Any product that may be evaluated in this article, or claim that may be made by its manufacturer, is not guaranteed or endorsed by the publisher.

## Supplementary Material

Supplementary Figure 1 Distinct immune characteristics underlined in periodontitis. **(A-C)** Human leukocyte antigen gene expression **(A)**, immune cell infiltration **(B)**, and immune pathway activities **(C)** between periodontitis (diseased) and healthy tissues. **P* < 0.05, ***P* < 0.01, ****P* < 0.001.
