# Supplementary material for: Identification of Key Pyroptosis-Related Genes and Distinct Pyroptosis-Related Clusters in Periodontitis
Source: Front Immunol. 2022 Jun 29;13:862049. doi: 10.3389/fimmu.2022.862049 (PMC9281553; doi:10.3389/fimmu.2022.862049)
Supplement: Supplementary file 2 [file Table_1.doc]

**Table S1 Detailed information of GSE10334 and GSE16134**

| **Dataset ID** | **Platform** | **Periodontitis** | **Normal** |
| --- | --- | --- | --- |
| GSE10334 | GPL570 | 183 | 64 |
| GSE16134 | GPL570 | 241 | 69 |
